# Geographical classification of malaria parasites through applying machine learning to whole genome sequence data

**DOI:** 10.1038/s41598-022-25568-6

**Published:** 2022-12-07

**Authors:** Wouter Deelder, Emilia Manko, Jody E. Phelan, Susana Campino, Luigi Palla, Taane G. Clark

**Affiliations:** 1 London School of Hygiene & Tropical Medicine, Keppel Street, London, WC1E 7HT UK; 2 Dalberg Advisors, 7 Rue de Chantepoulet, 1201 Geneva, Switzerland; 3 Department of Public Health and Infectious Diseases, University of Rome La Sapienza, Rome, Italy

**Keywords:** Population genetics, Machine learning

## Abstract

Malaria, caused by Plasmodium parasites, is a major global health challenge. Whole genome sequencing (WGS) of *Plasmodium falciparum* and *Plasmodium vivax* genomes is providing insights into parasite genetic diversity, transmission patterns, and can inform decision making for clinical and surveillance purposes. Advances in sequencing technologies are helping to generate timely and big genomic datasets, with the prospect of applying Artificial Intelligence analytical techniques (e.g., machine learning) to support programmatic malaria control and elimination. Here, we assess the potential of applying deep learning convolutional neural network approaches to predict the geographic origin of infections (continents, countries, GPS locations) using WGS data of *P. falciparum* (n = 5957; 27 countries) and *P. vivax* (n = 659; 13 countries) isolates. Using identified high-quality genome-wide single nucleotide polymorphisms (SNPs) (*P. falciparum*: 750 k, *P. vivax*: 588 k), an analysis of population structure and ancestry revealed clustering at the country-level. When predicting locations for both species, classification (compared to regression) methods had the lowest distance errors, and > 90% accuracy at a country level. Our work demonstrates the utility of machine learning approaches for geo-classification of malaria parasites. With timelier WGS data generation across more malaria-affected regions, the performance of machine learning approaches for geo-classification will improve, thereby supporting disease control activities.

## Introduction

Malaria, caused by *Plasmodium* parasites and transmitted by Anopheles mosquitoes, remains a pressing global health problem, with a mortality and morbidity burden heavily concentrated among children less than five years old. The morbidity and mortality impacts of *Plasmodium falciparum* malaria are predominantly concentrated in Sub-Saharan Africa, whereas the burdens of *Plasmodium vivax* are most heavily felt in Asia and South America^1^. The complex co-evolutionary history between *Plasmodium* parasites, humans, and Anopheles mosquitoes is contained within the genome of each organism, and genomic tools and data are of key importance for understanding the fundamental genetic underpinning of malaria, its geo-spatial distribution and control strategies to eliminate it. There is a rapidly growing number of *P. falciparum* and *P. vivax* isolate DNA that have undergone whole genome sequencing (WGS), with continued advances in genomic technologies likely to accelerate the timely generation of datasets from clinical and surveillance blood samples to inform disease epidemiology and control.

The rich information contained in WGS data can be used to infer transmission patterns, detect drug resistance, and support wider malaria control initiatives and elimination strategies^2,3^. WGS data in combination with population genomic methods can detect selective sweeps associated with drug resistance and infer the geographic origin of infections, including if infections are found to be imported or drug resistant and whether treatment should be adapted accordingly. It is known that malaria parasites have a population structure primarily based on geography^4,5^. Several informative molecular barcodes for speciation and geography have been developed^2,3^, but typically these barcodes have not used the whole genome due to the high-dimensionality of the data and the associated computational cost^3^. However, machine learning (a subfield of Artificial Intelligence) with its ability to incorporate and analyse very large and high-dimensional datasets in an efficient manner, seems potentially well suited for geo-predicting using WGS data. Machine learning can be applied for classification, which concerns predicting a label (e.g., country, continental region), and regression, which involves predicting a quantity (e.g., longitude or latitude).

Machine learning has been applied effectively across a variety of problems in malaria research, including the detection of evolutionary selection associated with drug resistance^6,7^, the classification and detection of parasites in red blood cells^8–11^, and antimalarial drug discovery^12^. Deep learning is a subset of machine learning where algorithms aim to extract and learn series of hierarchical representations, often leveraging large amounts of data. The application of deep learning, and especially neural networks, has been explored within population genetics^13,14^, including for other pathogens^15,16^. Pioneering work has also shown that machine learning, including deep learning convolutional neural networks (CNNs), can be used to predict geographic locations from human, mosquito and *P. falciparum* genetic variation^17^, building on methods and the use of large genotyping chips or WGS for population structure assessment^18,19^. Here, we aim to further expand on the application of geo-prediction for malaria parasites by using a very large dataset of isolates sourced globally, *(P. falciparum*, n = 5957, 27 countries; *P. vivax,* n = 659, 13 countries) across 11 regions (South East Asia (SEA), Southern SEA (SSEA), South Asia, South America, West Africa, Central Africa, South Central Africa, East Africa, Horn of Africa, Southern Africa, Oceania). We explore the potential of both regular machine learning approaches that aim to learn representations from sequence and geographical data, as well as deep learning approaches that aim to learn and extract layers of hierarchical representations of SNP combinations linked to geography. We compare four commonly applied approaches, including classification methods that predict locations and subsequently interpolate to specific coordinates, as well as compare the performance across geographies (countries) both including the observations within those and excluding them from the training sets used to develop the models.

## Materials and methods

### Processing of raw sequencing data

Publicly available raw Illumina (> 150 bp paired end) sequence data from previously published studies of *P. falciparum* and *P. vivax* was downloaded from the ENA repository (see S1 Table and S2 Table for accession numbers), and accompanied by meta-data including locations of sampling (see S1 Table and S2 Table for latitude and longitude coordinates). The data included public raw sequence and GPS data from MalariaGEN projects (www.malariagen.net). Raw WGS data for *P. falciparum* (n = 5957) and *P. vivax* (n = 659) were aligned with the *Pf3D7* (v3) and *PvP01* (v1) reference genomes, respectively, using bwa-mem software (v0.7.12) using default parameter settings (e.g., concerning mismatch and sequence read clipping penalties; see http://bio-bwa.sourceforge.net/bwa.shtml). The samtools (v1.9) functions fixmate and markdup were applied to the resulting BAM files to call a set of potential variants^20^. For variant quality control, calibration assessments were performed using the GATK’s BaseRecalibrator and ApplyBQSR functions, benchmarking off known high quality variants from genetic crosses for *P. falciparum*^5,21^ and previously curated datasets for *P. vivax*^20^. A revised set of SNPs and insertions/deletions (indels) was called with GATK’s HaplotypeCaller (version 4.1.4.1) using the option -ERC GVCF^5,22^. Variants were then assigned a quality score using GATK’s Variant Quality Score Recalibration (VQSR), and those with a VQSLOD score < 0, representing variants more likely to be false than true, were filtered out^7,22^. Additionally, SNPs were removed if they had more than 10% missing alleles^7,22^.The resulting dataset comprised of parasite genomes of *P. falciparum* (5,957 isolates, 750 k SNPs) and of *P. vivax* (659 isolates, 588 k SNPs). The population structure was assessed using a principal component analysis (PCA) of between isolate SNP differences. In parallel, ADMIXTURE analysis^23^ was performed to understand the composition of ancestral groups across geography, where the optimal number of groups (K) was established using cross validation with values ranging between 1 and 20. This cross validation analysis led to 10 ancestral groups for both *P. falciparum* and *P. vivax* (K = 10).

### Statistical models and performance

Using machine learning (ML) and deep learning (DL) statistical models, the goal was to use SNPs to predict geographical source at a location (GPS), country, and regional resolution. We applied two standard models for classification at a country and region level: (1) penalized multinomial logistic regression classifier (LOG-C; ML); (2) CNN (CNN-C; DL). Subsequently, we used the predictive probabilities placed on different locations to perform a weighted interpolation between these locations and make predictions at the GPS coordinate level.

In particular, the final prediction location (longitude and latitude) was determined by a weighted average of classifier predictions, where weights are the probabilities placed by the model on each location.

We also applied two regression models for GPS coordinate prediction: (iii) penalised linear regression model (LIN-R; ML); (iv) CNN (CNN-R; DL). The LOG-C and LIN-R models were tuned on the regularization strength C for the L1 penalty (LASSO) and implemented in the sklearn Python package (https://scikit-learn.org). The penalty parameters were tuned using cross-validation (see below, S3 Table). The deep learning CNN architecture was implemented using the Keras library (version 2.2.4)^24^ in Python. Our CNN models had an architecture with a soft-max prediction layer and regularization through dropout^25^ to prevent overfitting and support transferability. The main model had one convolutional layer with 4 filters, with respective filter size of (40, 9) followed by two drop-out and dense layers with ReLu activation (similar to^17^), and applied the Stochastic Gradient Descent algorithm for optimisation. We trained and validated the models for 1000 epochs. The parameterisation of the models is summarised (S3 Table). We created a stratified three-fold split in the dataset (80% training, 10% validation, 10% test) for all models, and used the validation dataset to cross-validate parameters (S3 Table). The LOG-C and LIN-R models were cross-validated (stratified, four-fold) on the regularization strength C for the L1 penalty. The reported scores (accuracy, mean weighted distance error) were calculated by making predictions on the hold-out test set (see S3 Table for the final parameter set). In addition, we conducted a “leave-one-geography-out”, where each single geography in the training dataset was omitted in turn, with the model trained on the remaining geographies, to understand generalizability towards previously unseen locations^26^.

Classification accuracy was determined after assigning predicted latitude and longitude pairs to individual countries. For the classification models, a mean (weighted) distance error was calculated using the Haversine method to allow for (angular) distance calculations along a sphere, based on the difference of the actual and estimated location. The latter was determined by a weighted average of classifier predictions, where weights are the probabilities placed by the model on each location. The accuracy was calculated based on the labels of the prediction versus the test data. In particular, the baseline accuracy using a naive prediction based on the most common country would be 18.8% for *P. falciparum* (Cambodia) and 24.3% for *P. vivax* (Thailand). For the regression models, the error was calculated using the Haversine method based on the difference between the predicted and actual latitude and longitude using angular distance.

## Results

### Malaria isolate sequence data and population structure

Raw WGS data with accompanying geographic origin information was available in the public domain for *P. falciparum* (n = 5957, 27 countries) and *P. vivax* (n = 659, 13 countries) (Table 1), which represent the global distributions for each parasite. Most *P. falciparum* isolates were sourced from SEA (2,648, 44.5%) followed by West Africa (2,042, 34.3%) and East Africa (451, 7.6%). Whilst, for *P. vivax,* most isolates were sourced from SEA (282, 42.9%) followed by South America (220, 33.4%) and SSEA (48) (Table 1). By analysing each species separately, high quality genome-wide SNPs were identified across the isolates (*P. falciparum* 750 k SNPs, *P. vivax* 588 k SNPs). Most SNPs have low minor allele frequencies (SNPs with MAF < 1%: *P. falciparum* 94.6%, *P. vivax* 77.6%) (S1 Figure). Most SNPs were in genic regions (*P. falciparum* 76.5%, *P. vivax* 54.3%), with a high proportion of non-synonymous (NS) amino acid changes (*P. falciparum* 63.0%, *P. vivax* 42.5%). The genetic diversity amongst *P. falciparum* isolates was relatively homogeneous across the 27 countries (SNP π: median 0.037, range 0.027–0.053), and lower in magnitude than *P. vivax*, whose data was sourced from 13 countries (SNP π: median 0.056, range 0.037–0.066) (Table 1).

**Table 1:** Sample origin and SNP Diversity by geographic location.

Region	Country	*Pf*. *SNP *Diversity	*Pf.* N*	*Pf.* %	*Pv. SNP *Diversity	*Pv.* N**	*Pv.* %
West Africa	Benin	0.040	76	1.3	–	–	–
	Burkina Faso	0.028	86	1.4	–	–	–
	Gambia	0.035	164	2.8	–	–	–
	Ghana	0.033	928	15.6	–	–	–
	Guinea	0.040	161	2.7	–	–	–
	Ivory Coast	0.034	70	1.2	–	–	–
	Mali	0.034	378	6.3	–	–	–
	Mauritania	0.035	77	1.3	–	–	–
	Nigeria	0.050	18	0.3	–	–	–
	Senegal	0.039	84	1.4	–	–	–
East Africa	Kenya	0.035	116	1.9	–	–	–
	Tanzania	0.035	320	5.4	–	–	–
	Uganda	0.053	15	0.3	–	–	–
Horn of Africa	Ethiopia	0.048	25	0.4	0.060	44	6.7
Central Africa	Cameroon	0.033	237	4.0	–	–	–
South Central Africa	DRC	0.032	339	5.7	–	–	–
Southern Africa	Madagascar	0.040	24	0.4	–	–	–
	Malawi	0.027	29	0.5	–	–	–
South Asia	India	–	–	–	0.062	40	6.1
	Bangladesh	0.037	83	1.4	–	–	–
South East Asia (SEA)	Cambodia	0.040	1118	18.8	0.049	70	10.6
	Laos	0.039	126	2.1	–	–	–
	Myanmar	0.039	246	4.1	0.061	27	4.1
	Thailand	0.038	928	15.6	0.056	160	24.3
	Vietnam	0.036	147	2.5	0.048	13	2.0
	China	–	–	–	0.066	12	1.8
Southern SEA (SSEA)	Malaysia	–	–	–	0.040	48	7.3
South America	Colombia	0.046	16	0.3	0.055	30	4.6
	Peru	0.037	24	0.4	0.059	88	13.4
	Brazil	–	–	–	0.061	82	12.5
	Mexico	–	–	–	0.039	20	3.0
Oceania	PNG	0.040	120	2.0	0.037	24	3.6
*Total*	–	–	*5955*	*100*	–	*658*	*100*

Pf *P. falciparum*, Pv *P. vivax*; PNG Papua New Guinea; DRC Democratic Republic of Congo.

**Figure 1:**
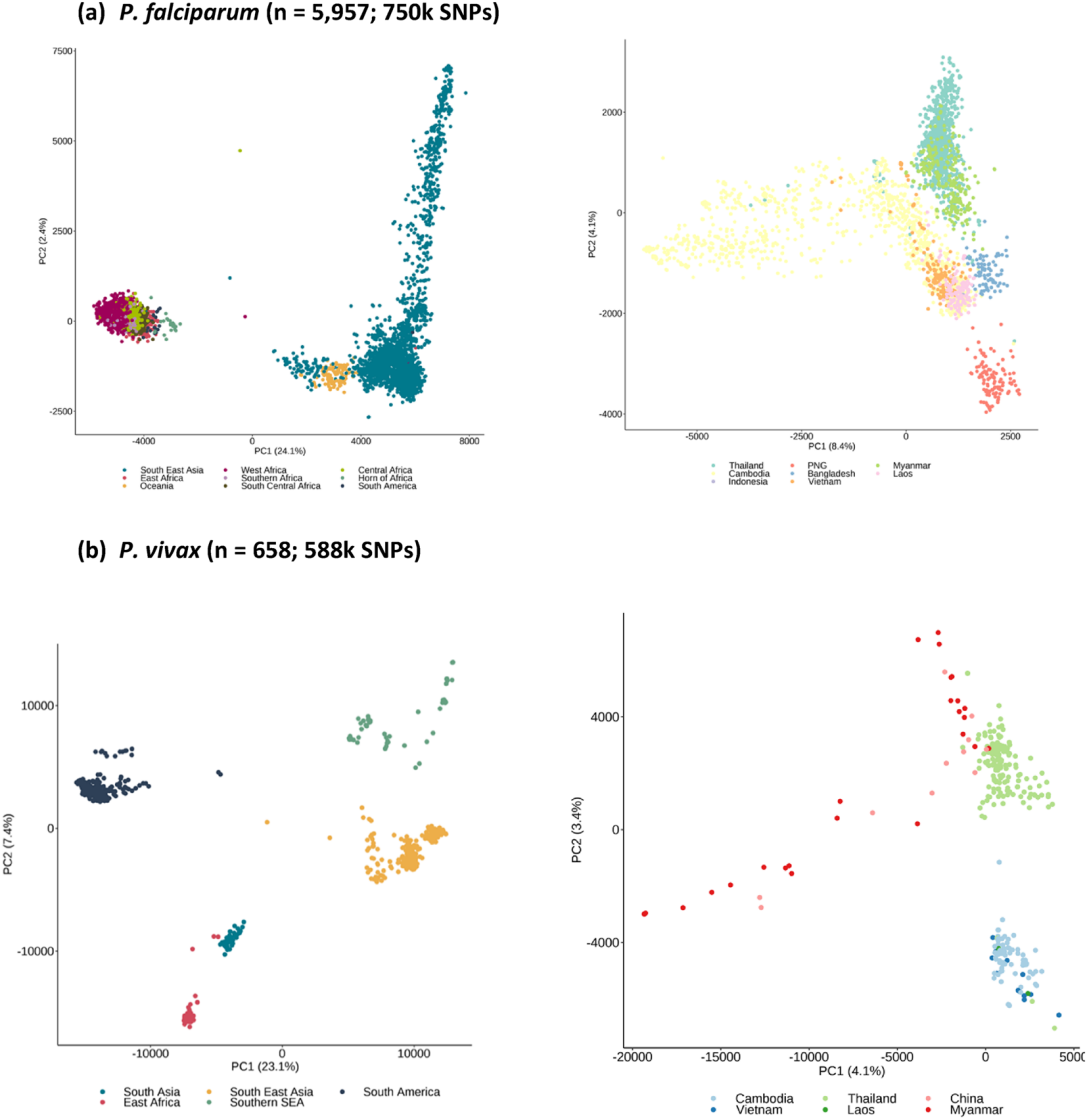
Population structure using principal component analysis based on all high-quality SNPs. Axes show percentage of variation explained by each principal component (PC).

**Figure 2:**
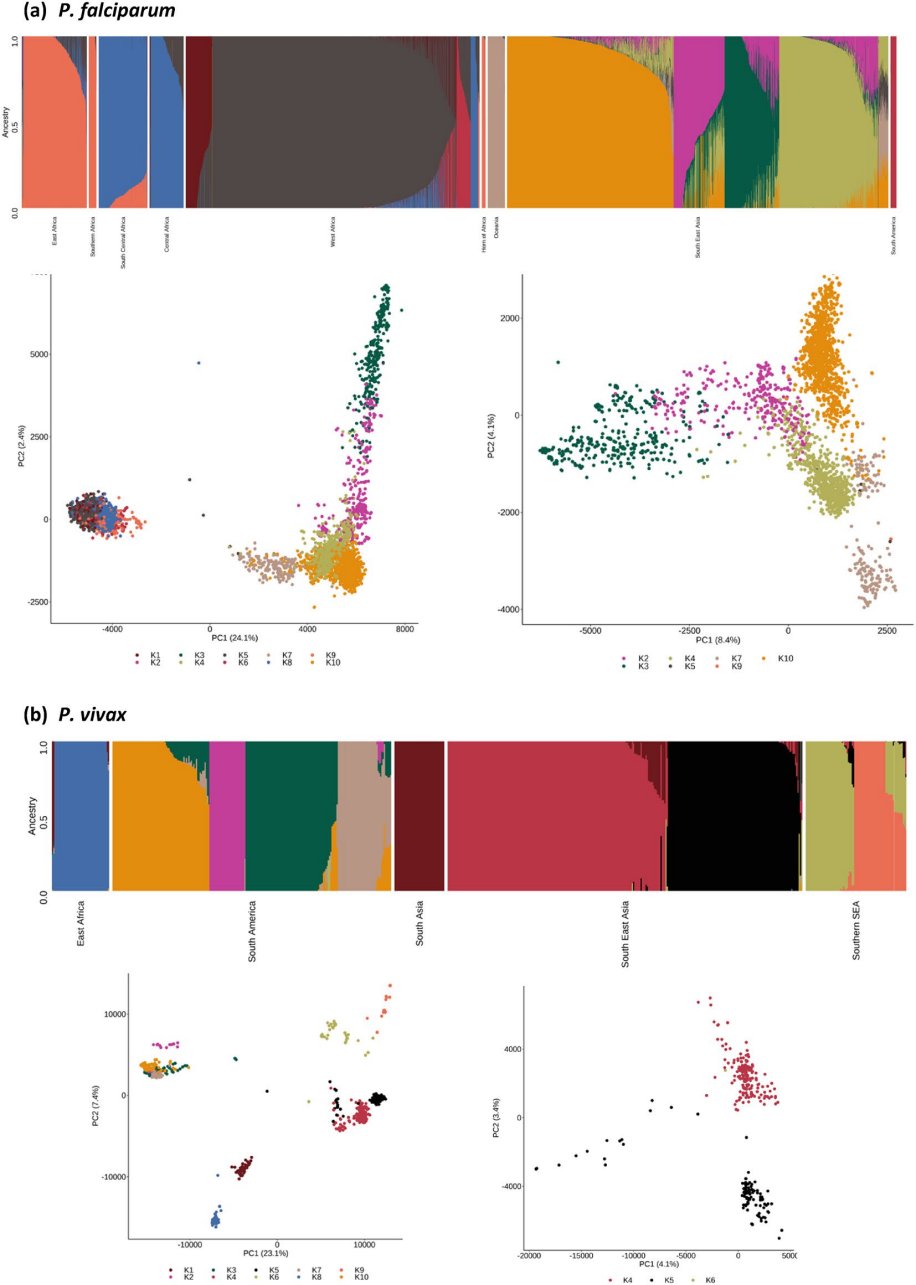
ADMIXTURE analysis involving 10 inferred ancestral populations (denoted as K1 to K10).

Unsupervised clustering methods were applied to the genome-wide SNPs of each species to reveal the extent of their population structure and linked (pseudo-)ancestral patterns. Principal component analysis (PCA) of *P. falciparum* and *P. vivax* isolates revealed the expected separation by continent, and clear evidence of population structure at both the regional and country level (Fig. 1). An analysis of population structure and ancestry using ADMIXTURE software^23^ determined the number of ancestral groups (*P. falciparum* K = 10, *P. vivax* K = 10), and their relative abundance for each isolate was estimated (Fig. 2). For *P. falciparum*, there were dominant ancestral groups across region and continent (Africa 4, SEA 4, Oceania 1, South America 1), with some evidence of mixture of ancestries (e.g., SEA isolates with 3 ancestral populations), but a general consistency within country. For *P. vivax*, the numbers of dominant ancestral groups by region differed from *P. falciparum* (South America 4, SEA 2, SSEA 2, East Africa 1, South Asia 1), due to sampling and Plasmodium species endemicity differences, such as the near absence of *P. vivax* in Africa. Overall, there was more homogeneity of ancestral groups within *P. vivax* isolates, with some groups broadly linked to neighbouring countries (comparison with Fig. 1). These analyses confirmed that spatial-genomic clustering and classification is possible using WGS data.

### Application of geo-classification models

**Figure 3:**
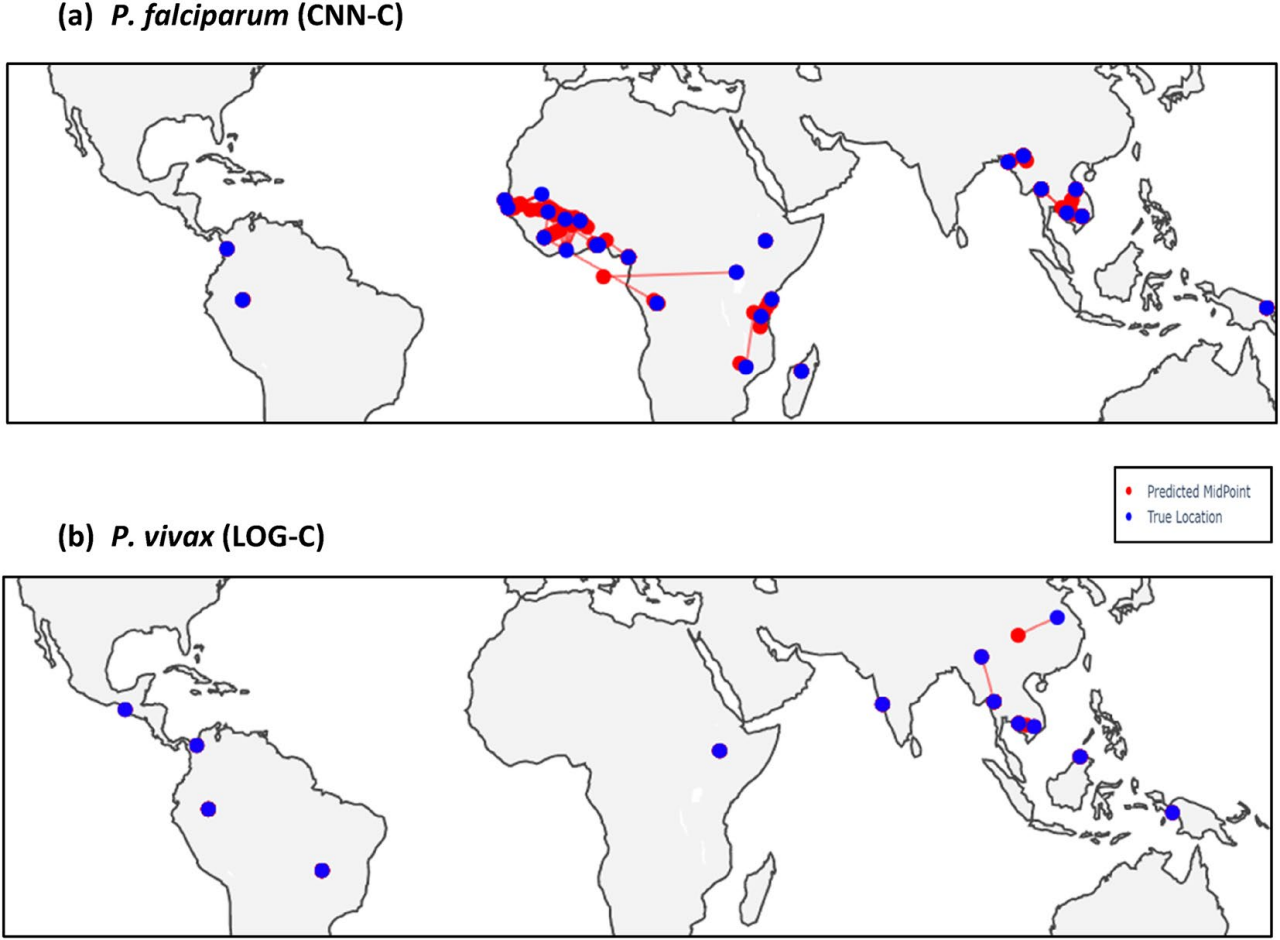
Maps with predicted vs. actual locations for the best predictive models. Blue points are the actual locations in the dataset, red points are the predicted locations (where different to actual), with red lines link the actual and the predicted locations. CNN-C deep learning Convolutional Neural Network classifier. LOG-C penalised multinomial logistic regression classifier.

For *P. falciparum,* the predictive performance of the classification methods (LOG-C, CNN-C) was stronger than for the regression models (LIN-R, CNN-R) in regional (Table 2) and country-wide (Table 3) analyses (mean distance error (km): LIN-R 470, LOG-C 93, CNN-R 245, CNN-C 77). For locations included in the training dataset, the performance of the classification models was close to 100% at the regional level, and close to 90% at the country level (S4 Table, S5 Table). The poorest performance of the models was for African populations, for example, the mean distance error for CNN-C was high in West African (267 km) and East African countries (117 km, especially Kenya and Uganda), as well as Malawi (530 km) (Table 3), compared to other regions. This observation is consistent with the complex ancestries in African populations (Fig. 2), as well as another deep learning analysis^17^. As expected, where we predicted countries absent in data used by the training models, the distance errors (km) were at least ~ five-fold larger (LIN-R 2246, LOG-C 1848, CNN-R 1983, CNN-C 1540), with the poorest predictions for Peru (Table 4). The best performing model in this setting was the CNN-C classifier (Fig. 3).

**Table 2:** Mean distance Error (km) per model by region using geographies included in the training data.

Parasite	Region	N	LIN-R*	LOG-C*	CNN–R	CNN –C*
Pf	West Africa	2042	665 [375–1354]	302 [5–681]	368 [161–1169]	267 [45–728]
	East Africa	451	708 [693–1198]	200 [3–1581]	297 [289–856]	117 [0–1856]
	Horn of Africa	25	569 [569–569]	0 [0–0]	124 [124–124]	0 [0–0]
	Central Africa	237	635 [635–635]	29 [29–29]	184 [184–184]	0 [0–0]
	SC Africa	339	478 [478–478]	3 [3–3]	34 [34–34]	0 [0–0]
	Southern Africa	53	490 [490–968]	7 [7–433]	1543 [1018–1543]	0 [0–530]
	SEA	2648	312 [247–744]	19 [8–121]	152 [39–559]	7 [0–53]
	South America	40	1936 [1820–2053]	3 [0–7]	3683 [2535–4832]	0 [0–0]
	Oceania	120	488 [488–488]	0 [0–0]	697 [697–697]	0 [0–0]
Pv	Horn of Africa	44	334 [334–334]	0 [0–0]	142 [142- 142]	0 [0–0]
	South Asia	40	500 [500–500]	0 [0–0]	517 [517–517]	0 [0–0]
	South East Asia	282	616 [156–2751]	25 [0–1033]	578 [288–704]	0 [0–1463]
	Southern SEA	48	213 [213–213]	0 [0–0]	957 [957–957]	0 [0–0]
	South America	220	906 [134–3080]	0 [0–0]	667 [574–2773]	0 [0–0]
	Oceania	24	175 [175–175]	0 [0–0]	1103 [1103–1103]	0 [0–0]

Pf *P. falciparum*, Pv *P. vivax,* * mean [range], CNN Convolutional Neural Network, SC South Central, SEA South East Asia; LOG-C multinomial logistic regression classifier; CNN-C CNN classifier; LIN-R penalised linear regression model; CNN-R CNN regression model.

**Table 3:** Mean distance error (km) per model on test data using those countries included in the training data.

Parasite	Region	Location	LIN-R	LOG-C	CNN-R	CNN-C
*P. falciparum*	West Africa	Benin	700	4	354	45
		Burkina Faso	374	96	161	88
		Gambia	775	132	317	107
		Ghana	401	48	193	52
		Guinea	751	515	459	402
		Ivory Coast	630	681	695	728
		Mali	563	345	208	271
		Mauritania	615	676	382	410
		Nigeria	1039	329	1169	329
		Senegal	1354	274	565	263
	East Africa	Kenya	693	200	297	117
		Tanzania	707	3	289	0
		Uganda	1198	1581	856	1856
	Horn of Africa	Ethiopia	568	0	124	0
	Central Africa	Cameroon	635	28	184	0
	SC Africa	DRC	477	2	34	0
	Southern Africa	Madagascar	490	6	1543	0
		Malawi	968	432	1018	530
	SEA	Bangladesh	743	9	159	0
		Cambodia	312	18	112	21
		Laos	276	121	152	53
		Myanmar	360	10	559	0
		Thailand	247	7	39	7
		Vietnam	356	90	199	0
	South America	Colombia	2052	0	4832	0
		Peru	1820	7	2535	0
	Oceania	PNG	488	0	697	0
	*Mean*		*470*	*93*	*245*	*77*
*P. vivax*	Horn of Africa	Ethiopia	334	0	142	0
	South Asia	India	500	0	517	0
	SEA	Cambodia	638	25	648	0
		China	2751	1033	704	1463
		Myanmar	616	311	350	311
		Thailand	604	0	288	0
		Vietnam	156	0	578	0
	SSEA	Malaysia	213	0	957	0
	South America	Brazil	3080	0	2773	6
		Colombia	1057	0	667	0
		Mexico	134	0	1502	0
		Peru	755	0	574	0
	Oceania	PNG	175	0	1103	0
	*Mean*		*890*	*33*	*819*	*36*

DRC Democratic Republic of Congo; PNG Papua New Guinea; CNN Convolutional Neural Network; LOG-C multinomial logistic regression classifier; CNN-C CNN deep learner classifier; LIN-R penalised linear regression model; CNN-R Penalised CNN regression model; SC South Central; SEA South East Asia; SSEA Southern SEA.

**Table 4:** Mean distance error (km) per model on test data for unseen geographies.

Parasite	Location	LIN-R	LOG-C	CNN-R	CNN-C
*P. falciparum*	Cambodia	496	669	322	628
	Cameroon	959	1545	1472	1636
	DRC	1150	2331	2531	2456
	Ethiopia	1118	1760	1252	1394
	Myanmar	703	731	470	728
	Peru	9050	4050	5856	2400
	*Mean*	*2246*	*1848*	*1983*	*1540*
*P. vivax*	Cambodia	591	323	1709	564
	Ethiopia	2499	5174	3528	4140
	Malaysia	459	1594	3617	2064
	Peru	2376	2943	1196	2852
	*Mean*	*1481*	*2508*	*2512*	*2405*

CNN Convolutional Neural Network; DRC Democratic Republic of Congo; LOG-C multinomial logistic regression classifier; CNN-C CNN deep learning classifier; LIN-R penalised linear regression model; CNN-R Penalised CNN regression model.

For *P. vivax*, the predictive performance of the classification methods (LOG-C, CNN-C) was also superior compared to regression models (LIN-R, CNN-R) across regional (Table 2) and country-wide (Table 3) analyses (mean distance error (km): LIN-R 890, LOG-C 33, CNN-R 819, CNN-C 36) (Table 3). For locations included in the training dataset, the performance of the classification models was close to 100% at both the regional and country level, with the poorest performance in neighbouring China and Myanmar (S4 Table, S5 Table). The (mean) distance error for the countries not used in the development of the model is distinctively larger (km: LIN-R 1481, LOG-C 2508, CNN-R 2512, CNN-C 2405), with the poorest predictions for Ethiopia and Peru (Table 4). The best performing model in this setting was a LIN-R regression (Fig. 3).

## Discussion

WGS data of *Plasmodium* parasites can detect imported infections, drug resistance, and transmission patterns, thereby assisting decision making in clinical and malaria control settings. With the implementation of WGS gaining traction across health systems, there is an opportunity to implement statistical learning methodologies to assist surveillance activities. A clear use-case includes the determination of the geographical origin of isolates, building on insights from previous work which shows that genomic data can be used to cluster parasites by geography^2–5^. Our work reveals that machine learning approaches, particularly those focusing on classification (e.g., deep learning CNNs), have the potential to accurately predict geographic locations at a GPS and country-level resolution. As expected, the performance was much stronger for isolates of which the geographic origin was already represented at the country level in the dataset, demonstrating the need for WGS to be implemented more widely to fill country gaps in genetic diversity. The weakest predictions were for *P. falciparum* in West and East Africa, where common ancestries, mixed infections, movement of people, drug resistance and malaria endemicities can complicate genetic diversity analysis. The distance errors are similar to a previous machine learning analysis of *P. falciparum* (median < 20 km), which implemented a single deep learning approach on a smaller dataset^17^. Our CNN for classification approach appeared to perform well across parasite species, was implemented with measures to minimise the effects of over-fitting, and its performance is likely to improve with greater isolate sampling and WGS data.

Whilst we have implemented a limited set of machine learning methods, there is scope to test alternative approaches (e.g., gradient boosted trees, support vector machines)^16^ or further optimise our model parametrisations (beyond the default settings) to improve performance. For example, while L1-penalized regression approaches are generally quite competitive, stability selection on top of the LASSO leads generally to improvements^27^. Moreover, the resulting model is white box and leads to a set of interpretable SNPs. CNNs are the most utilised deep learning network type, and known to outperform alternative approaches^28^. However, one limitation of CNN models is their “black box” nature, with a complex architecture consisting of several layers, and in our context (and others^17^) making it difficult to establish which (combinations of) SNPs are informative for the geographical profiling. Other studies have used population genomic approaches to determine informative SNPs, with a focus on applying genotyping assays or amplicon sequencing for resource poor settings^2,3^. We provide computer code to implement the models, to assist future assessments in simulation or empirical studies. Future work should focus on the development of an online “geo-locator” tool that reveals a prediction of location, which can be assessed for its plausibility against the actual position, if known, and feedback into the model building and learning process. Such a framework could also be extended to integrate explicit drug resistance markers^29^, as well as genomic data for malaria vectors^17^, and use sequences generated on portable and field deployable sequencing platforms (e.g., Oxford Nanopore Technology MinION). Such tools would be of immediate value to malaria control programs in endemic countries, including those that are implementing elimination activities who wish to differentiate between locally acquired or imported infections. It would also assist those countries with low malaria burden, including through the detection of imported parasites that could threaten malaria elimination targets.

In summary, our study has demonstrated that machine learning methods can play an informative role in determining the geographic origin of WGS isolates, thereby providing important insights for both control and surveillance activities. Further, such approaches will be scalable when WGS becomes routine and cost effective, resulting in a setting with increasingly “big data” being available for decision making. The utility of this “learning” system will improve with time, as underlying methodologies and model performances improve with more data becoming available, and they are implemented within informatic tools to assist surveillance and clinical decision making. This utility underscores the benefit of making sequencing data and linked geographical information publicly available to global databases in a more-timely fashion to understand infection dynamics, the advantages of which have also been demonstrated by the COVID-19 crisis.

## Conclusion

Advances in sequencing technologies are making real time genomics-informed surveillance and clinical management a reality. With the resulting big genomic datasets, our study has shown that machine learning methods, a subset of Artificial Intelligence, can accurately predict the geographical source of malaria parasites from sequence data. With greater geographical coverage and informatics infrastructure, such approaches will improve in performance and assist malaria control and elimination activities.

## Supplementary Information

Supplementary Information 1.

Supplementary Information 2.

Supplementary Information 3.

Supplementary Information 4.

## Data Availability

The raw WGS data is available from the European Nucleotide Archive (ENA) (see S1 Table and S2 Table for project accession numbers). Computing code and machine learning models are available from https://github.com/WDee/GeoComparison.
