# Supplementary material for: Geographical classification of malaria parasites through applying machine learning to whole genome sequence data
Source: Sci Rep. 2022 Dec 7;12:21150. doi: 10.1038/s41598-022-25568-6 (PMC9729610; doi:10.1038/s41598-022-25568-6)
Supplement: Supplementary file 4 — Supplementary Information 4. [file 41598_2022_25568_MOESM4_ESM.docx]

**S1 Table**

Please note the separate file S1_Table.xlsx

**S2 Table**

Please note the separate file S2_Table.xlsx

**S3 Table**

**The machine learning parameter settings for the models**

| **Name** | **Classifier** | **Predicts** | **Fixed Parameters** | **Cross-validated parameters *** |
| --- | --- | --- | --- | --- |
| LOG-C** | Penalised Multinomial Logistic Regression – classification | Region, Country, and GPS | Penalty type = ”L1”  Tolerance=0.001  Maximum iterations=1000 | Penalty = 0.01/0.1 |
| LIN-R** | Penalised Linear Regression -regression | GPS | Penalty type = ”L1”  Tolerance=0.001  Maximum iterations=1000 | Penalty = 0.003/0.003 |
| CNN-C | Convolutional Neural Network -classification | Region, Country, and GPS | Epochs=1000  Early stopping with patience of 900 | - |
| CNN-R | Convolutional Neural Network – regression | GPS | Epochs=1000  Early stopping with patience of 900 | - |

GPS Global Positioning System; LOG-C penalised multinomial logistic regression classifier; CNN-C CNN classifier; LIN-R penalised linear regression model; CNN-R CNN regression model.

* Performed on *P. falciparum* and *P. vivax* data separately, across a cross-validation range of parameter values of 0.001, 0.0031, 0.01, 0.031, 0.1, 0.31 and 1, resulting in this case in the same penalty values for *P. falciparum* and *P. vivax*.

** There are two penalty parameters due to latitude/longitude

**S4 Table**

**Classification accuracy at a country level for *P. falciparum* (Pf) and *P. vivax* (Pv)**

| **Region** | **Country** | **Pf**  **LOG-C** | **Pf**  **CNN-C** | **Pv**  **LOG-C** | **Pv**  **CNN-C** |
| --- | --- | --- | --- | --- | --- |
| West Africa | Benin | 100 | 100 | - | - |
|  | Burkina Faso | 75.0 | 75.0 | - | - |
|  | Gambia | 93.8 | 100.0 | - | - |
|  | Ghana | 95.7 | 90.3 | - | - |
|  | Guinea | 62.5 | 62.5 | - | - |
|  | Mali | 63.2 | 63.2 | - | - |
|  | Mauritania | - | 50.0 | - | - |
|  | Nigeria | 50.0 | 50.0 | - | - |
|  | Senegal | 62.5 | 50.0 | - | - |
| East Africa | Kenya | 63.6 | 72.7 | - | - |
|  | Tanzania | 100 | 100 | - | - |
|  | Uganda | 50.0 | 50.0 | - | - |
| Horn of Africa | Ethiopia | 100 | 100 | 100 | 100 |
| Central Africa | Cameroon | 95.7 | 100 | - | - |
| South Central Africa | DRC | 100 | 100 | - | - |
| Southern Africa | Madagascar | 100 | 100 | - | - |
|  | Malawi | 66.7 | 33.3 | - | - |
| South Asia | India | - | - | 100 | 100 |
|  | Bangladesh | 100 | 100 | - | - |
| South East Asia (SEA) | Cambodia | 98.2 | 97.3 | 100 | 100 |
|  | Laos | 83.3 | 83.3 | - | - |
|  | Myanmar | 100.0 | 95.8 | 66.7 | 66.7 |
|  | Thailand | 98.9 | 98.9 | 100 | 100 |
|  | Vietnam | 71.4 | 92.9 | 100 | 100 |
|  | China | - | - | 0 | 100 |
| Southern SEA | Malaysia | - | - | 100 | 100 |
| South America | Colombia | 100 | 100 | 100 | 100 |
|  | Peru | 100 | 100 | 100 | 100 |
|  | Brazil | - | - | 100 | 100 |
|  | Mexico | - | - | 100 | 100 |
| Oceania | Papua New Guinea | 100 | 100 | 100 | 100 |

CNN Convolutional Neural Network, DRC Democratic Republic of Congo; LOG-C multinomial logistic regression classifier; CNN-C CNN deep learning classifier; LIN-R penalised linear regression model; CNN-R Penalised CNN regression model.

**S5 Table**

**Confusion matrices for the best predictive classification models**

1. **­ *P. falciparum* (CNN-C, regional level)**

Please note the separate file S5_Table.xlsx

1. ***P. vivax* (LOG-C, regional level)**

Please note the separate file S5_Table.xlsx

1. **­ *P. falciparum* (CNN-C, country level)**

Please note the separate file S5_Table.xlsx

1. ***P. vivax* (LOG-C, country level)**

Please note the separate file S5_Table.xlsx

CNN Convolutional Neural Network, LOG-C multinomial logistic regression classifier; CNN-C CNN deep learning classifier

**S1 Figure**

**Distribution of the minor allele frequencies across the SNPs**

1. ***P. falciparum* (N=750k SNPs)**

***
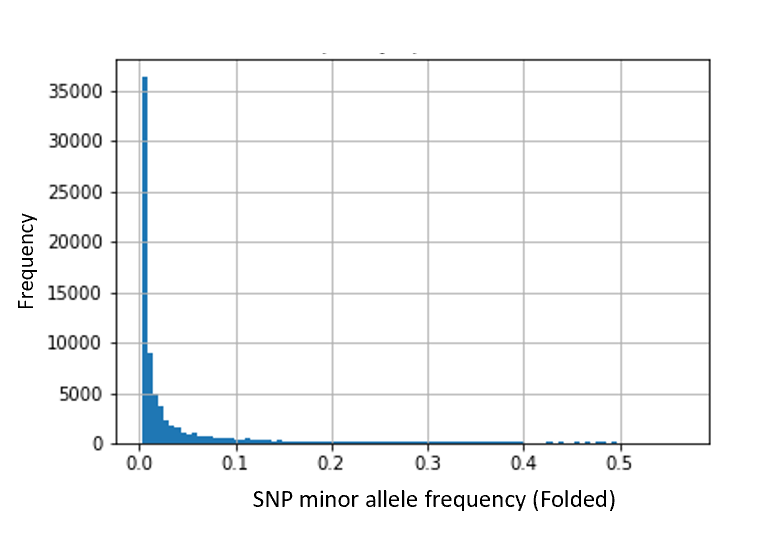
***

1. ***P. vivax* (N=588k SNPs)**

***
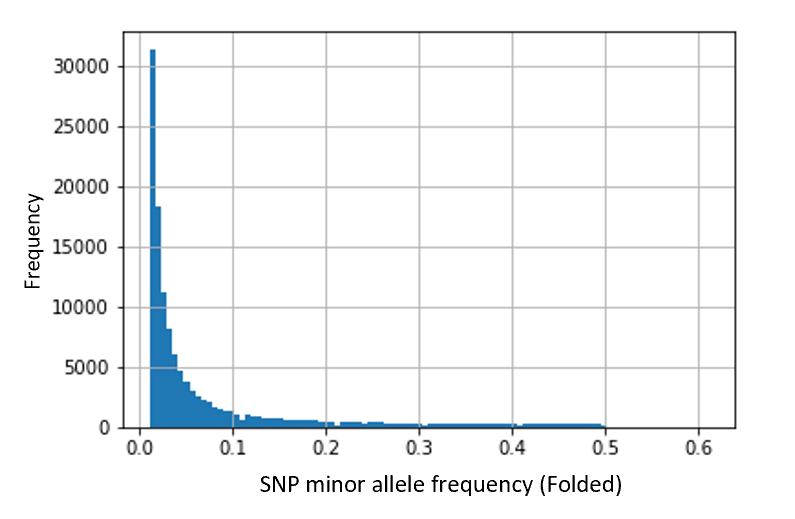
***

**S2 Figure**

**Maps with predicted vs. actual locations for all models**

1. ***P. falciparum***

***
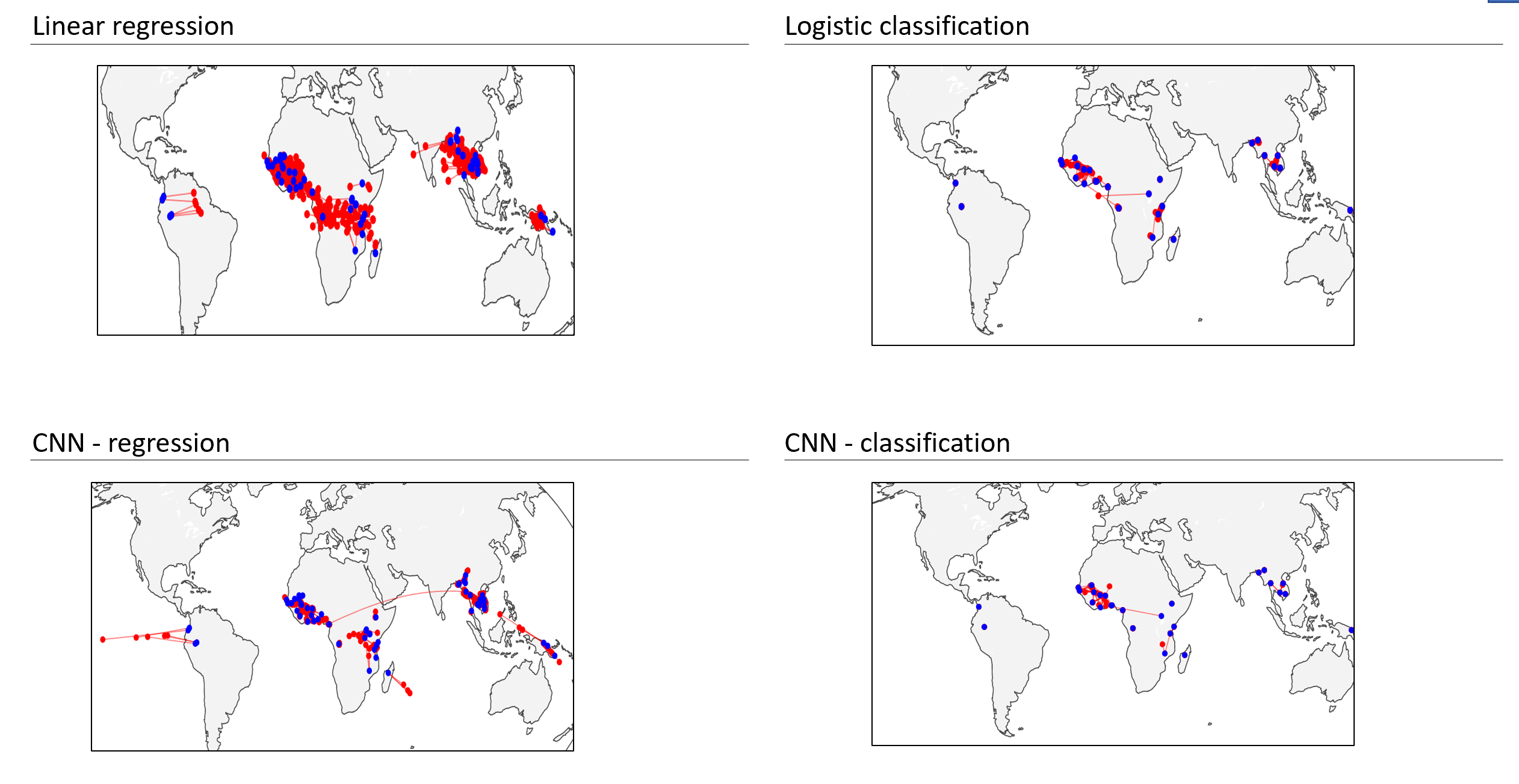
***

Legend: Blue points are the actual locations in the dataset, red points are the predicted locations, with red lines linking the actual and the predicted locations. Logistic classification refers to a multinomial logistic model.

1. ***P. vivax***

**
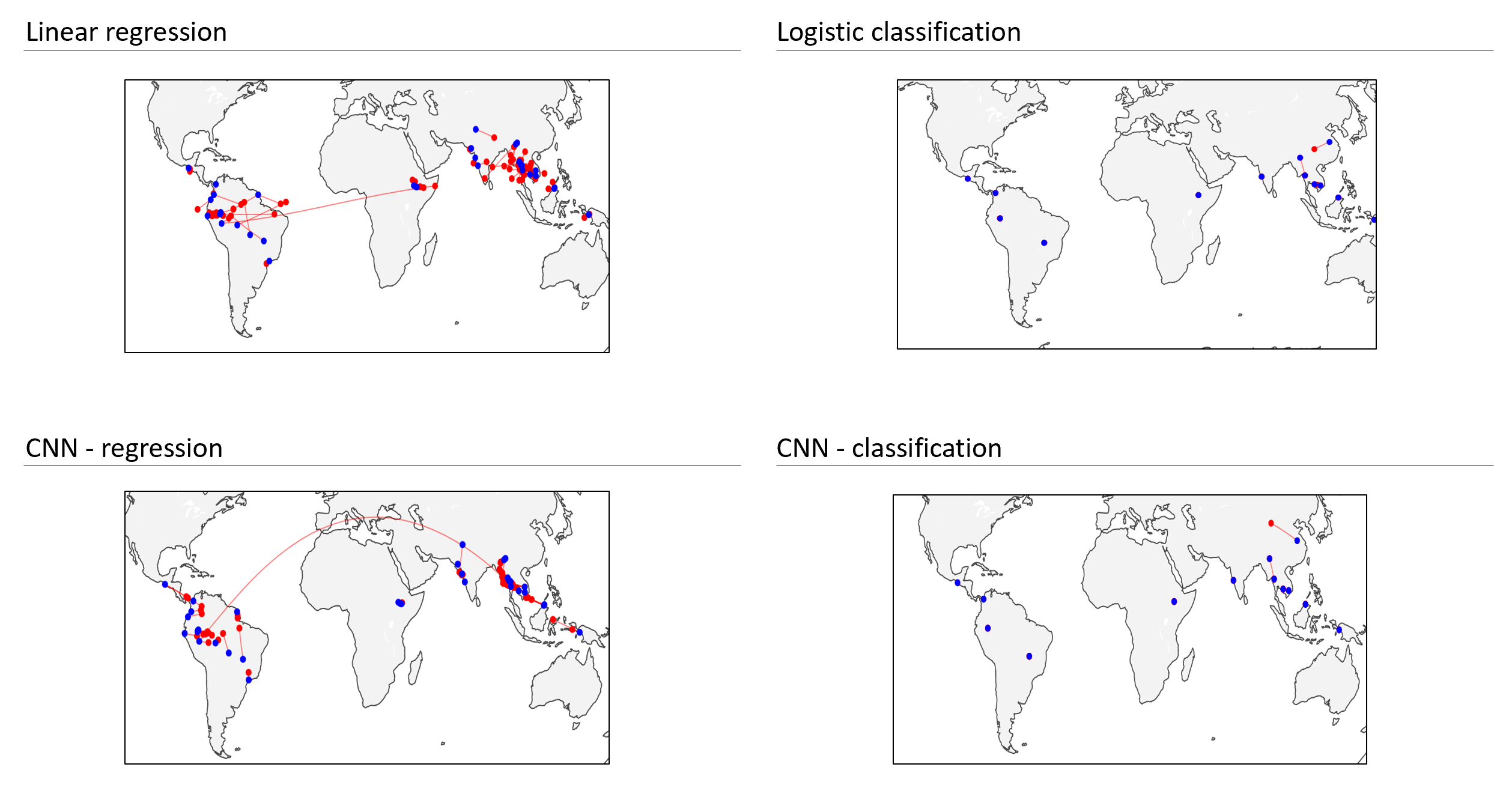
**

Legend: Blue points are the actual locations in the dataset, red points are the predicted locations, with red lines linking the actual and the predicted locations; Logistic classification refers to a multinomial logistic model.
